# Suppression by Thimerosal of *Ex-Vivo* CD4^+^ T Cell Response to Influenza Vaccine and Induction of Apoptosis in Primary Memory T Cells

**DOI:** 10.1371/journal.pone.0092705

**Published:** 2014-04-01

**Authors:** Emily Loison, Béatrice Poirier-Beaudouin, Valérie Seffer, Audrey Paoletti, Vered Abitbol, Eric Tartour, Odile Launay, Marie-Lise Gougeon

**Affiliations:** 1 Antiviral Immunity Biotherapy and Vaccine Unit, Institut Pasteur, Paris, France; 2 Inserm U1030, Institut Gustave Roussy, Villejuif, France; 3 Gastroenterology Department, Hôpital Cochin, AP-HP, Paris, France; 4 Inserm U970, Université Paris Descartes, PARCC/HEGP, Paris, France; 5 Centre d’Investigation Clinique BT-505, Hôpital Cochin, AP-HP, Paris, France; University of Iowa, United States of America

## Abstract

Thimerosal is a preservative used widely in vaccine formulations to prevent bacterial and fungal contamination in multidose vials of vaccine. Thimerosal was included in the multidose non-adjuvanted pandemic 2009 H1N1 vaccine Panenza. In the context of the analysis of the *ex-vivo* T cell responses directed against influenza vaccine, we discovered the *in vitro* toxicity Panenza, due to its content in thimerosal. Because thimerosal may skew the immune response to vaccines, we investigated in detail the *ex-vivo* effects of thimerosal on the fate and functions of T cells in response to TCR ligation. We report that *ex-vivo* exposure of quiescent or TCR-activated primary human T cells to thimerosal induced a dose-dependent apoptotic cell death associated with depolarization of mitochondrial membrane, generation of reactive oxygen species, cytochrome c release from the mitochondria and caspase-3 activation. Moreover, exposure to non-toxic concentrations of thimerosal induced cell cycle arrest in G0/G1 phase of TCR-activated T cells, and inhibition of the release of proinflammatory cytokines such as IFN gamma, IL-1 beta, TNF alpha, IL-2, as well as the chemokine MCP1. No shift towards Th2 or Th17 cells was detected. Overall these results underline the proapoptotic effect of thimerosal on primary human lymphocytes at concentrations 100 times less to those contained in the multidose vaccine, and they reveal the inhibitory effect of this preservative on T-cell proliferation and functions at nanomolar concentrations.

## Introduction

Thimerosal is a preservative used widely in vaccine formulations to prevent bacterial and fungal contamination in multidose vials of vaccine [1] [2]. Thimerosal, named also thiomersal or merthiolate in clinical studies, is an ethylmercury-containing pharmaceutical compound that contains 49.6% mercury by weight and metabolizes into ethylymercury (etHg) and thiosalicylate [3]. Thimerosal has served as a preservative in vaccines since 1930, but in the late 1990 concerns came as more thimerosal-containing vaccines were added to the recommended infant and child immunization schedule [4]. Research on the specific *in vivo* toxicity of low doses of etHg relevant to vaccines has only recently been performed [5] [6], [7]. *In vitro*, thimerosal has been shown to cause a number of neurotoxic changes, including neuronal mitochondrial cell death characterized by the release of cytochrome c and apoptosis inducing factor from mitochondria to cytosol [8], DNA breaks and caspase 3 activation in cultured human neuronal cells [9], and mitochondrial-mediated apoptosis in a human neuroblastoma cell line following exposure to μM concentrations of thimerosal [10]. The deleterious effects of thimerosal were also reported on HeLa S epithelial cells, inducing an oxidative stress and cell death that were completely suppressed by pretreating the cells with N-acetyl-l-cysteine (NAC), a radical scavenger [11]. Thimerosal could also cause S phase arrest followed by mitochondrial apoptosis in murine myoblast cells that occurred via inhibition of the PI3K/Akt/survivin signaling pathway [12]. Surprisingly, little is known about the impact of thimerosal on immune cell functions. Trompezinski *et al*. found that it induced an oxidative stress in monocyte-derived dendritic cells [13], and Agrawal et al. reported that thimerosal could exercise a Th2 promoting effect through modulation of functions of dendritic cells [14]. At the T cell level, thimerosal was reported to induce caspase-dependent mitochondrial apoptosis in the human leukemic Jurkat T cells [15], [16].

In the context of the clinical trial MICIVAX, designed to compare the efficacy and safety of influenza vaccine in patients with inflammatory bowel disease (IBD) receiving immunosuppressive therapy with patients not receiving immunosuppressants, we monitored the *ex-vivo* T cell response directed against the multidose non-adjuvanted pandemic 2009 H1N1 vaccine Panenza. We found that Panenza was toxic when used *ex-vivo* on patients' peripheral blood lymphocytes (PBMC) in T-cell assays, and this multidose vaccine-related toxicity was attributed to the preservative thimerosal. Because thimerosal may skew the immune response to vaccines, we investigated in detail the *ex-vivo* effects of thimerosal on the fate and functions of T cells in response to TCR ligation.

## Material and Methods

### Vaccines and Antigens

All the following vaccines, except Pandemrix, were obtained from Sanofi-Pasteur MSD (Lyon, France). Mutagrip (0.5 ml/dose) contains Hemagglutinin (HA) and Neuraminidase (N) proteins from the following three influenza strains (A/Brisbane/59/2007 [H1N1]-like, A/Brisbane/10/2007 [H3N2], B/Brisbane/60/2008-like). Each dose includes 15 μg of the various HA proteins but no thimerosal. Panenza, in its multidose format (10 doses), contains for each dose 15 μg of HA derived from the A/California/7/2009 [H1N1]- like strain and 45 μg of thimerosal.

Pandemrix from GlaxoSmithKline (Marly-le-Roi, France) contains for each dose 3.5 μg of HA derived from the A/California/7/2009 [H1N1]-like strain, the AS03 adjuvant and 5 μg of thimerosal.

PepTivator-CMV pp65, PepTivator-CMV IE1, PepTivator-EBV EBNA-1 and PepTivator-EBV BZLF (Miltenyi Biotec SAS, Paris, France) were used at 0.25 μg/ml, EBV, Tetanus toxoid (TT) and tuberculin PPD (Statens Serum Institut, Copenhagen, Denmark) were used at 5 μg/ml. As positive control, PBMC were stimulated with plate-bound anti-CD3 (1 μg/ml) and anti-CD28- mAbs (2 μg/ml) (Miltenyi Biotec SAS, Paris, France) during 1 to 3 days, according to the experiments.

Thimerosal was purchased from Sigma-Aldrich (St Quentin-Fallavier, France) and diluted in sterile water to obtain a 1 g/ml stock solution.

### Study Design

Human peripheral blood mononuclear cells (PBMCs) were isolated from heparinized blood of healthy adult donors (HD) provided by the Etablissement Français du Sang (EFS, Paris) in the setting of EFS-Institut Pasteur convention, or from subjects vaccinated with Mutagrip or Panenza. Some of these subjects were enrolled in the clinical trial MICIVAX. The study was approved under the authorization number 2704 by the “Ile-de France III” Ethics Committee, Hôpital Tarnier-Cochin, Paris, France. It was designed to detect T cell responses directed against seasonal and pandemic influenza 2009 H1N1 vaccine in subjects with inflammatory bowel disease. All the donors gave written informed consent for samples to be used in this study, and all samples and data were anonymized.

### Flow Cytometry Assays

#### Membrane staining

The following conjugated monoclonal antibodies (mAbs) were used: anti-CD3(SK7)-FITC, –PE or -APC, anti-CD4(SK3)-FITC or –PerCP, anti-CD8(SK1)-FITC or –PerCP, anti-CD19(SJ25C1)-PE, anti-CD14(M5E2)-FITC, anti-CD56(NCAM16.2)-APC, anti-CD16(3G8)-FITC, all purchased from Becton Dickinson (Le Pont de Claix, France). Cells were stained for surface markers (at 4°C in the dark for 30 min) using mixtures of mAbs diluted in PBS containing 0.5% BSA and 0.01% NaN3 (PBA). Appropriate isotype control mAbs were used for each staining combination. Samples were acquired on a FACSCalibur (BD) flow cytometer using BD Cell Quest Pro™ software, and analyzed using FlowJo Software (Tree Star, Inc).

#### CFSE staining

PBMC were stained with 1 μM carboxyfluorescein succinimidyl ester (CFSE) (CellTrace cell prolifération kit; Molecular Probes/Invitrogen) in PBS for 8 min at 37°C at a concentration of 10^7^ cells/ml. The labeling was stopped by washing the cells twice with RPMI 1640 culture medium containing 10% FBS. The cells were then resuspended at the desired concentration and subsequently used for proliferation assays, as previously reported [17].

#### Apoptosis assay using 7-AAD (7-amino-actinomycin D) staining

Apoptosis of unstimulated or stimulated PBMCs, CFSE-labeled or unlabeled, was determined using the 7-AAD assay, as we previously reported [18] [19]. Briefly, cultured cells were stained with 20 μg/ml nuclear dye 7-AAD (Sigma-Aldrich) for 30 min at 4°C, washed in PBA and fixed in PBA 1% PFA. Samples were acquired on a FACSCalibur (BD) flow cytometer using Summit software (Beckman Coulter), and analyzed using FlowJo Software. FSC/7-AAD dot plots distinguish living (FSC^high^/7-AAD^neg^) from apoptotic (FSC^high^/7-AAD^+^) cells, apoptotic bodies (FSCl^ow^/7-AAD^+^) and debris (FSC^low^/7-AAD^neg^).

#### Cell cycle analysis

The impact of thimerosal on cell cycling was determined with the combined staining with BrdU and 7-AAD (BD Pharmingen BrdU flow kit) that permits the enumeration and characterization of cells that are actively synthesizing DNA (BrdU incorporation) in terms of their cell cycle position (ie, G0/1, S, or G2/M phase) defined by 7-AAD staining intensities. Staining was performed according to the manufacturer's recommendations.

#### ΔY_m_ analysis

Variations of the mitochondrial transmembrane potential ΔY_m_ during thimerosal-induced T-lymphocyte apoptosis were studied using 3,3′-dihexyloxacarbocyanine iodide (DiOC6(3), Invitrogen). This cyanine dye accumulates in the mitochondrial matrix under the influence of the ΔY_m_. Staining conditions were previously described [20]. Briefly, 2.5 10^5^ stimulated PBMCs were incubated in 100 μl of PBS containing 0.1 μM of DiOC6(3) for 30 min. DiOC6(3) membrane potential-related fluorescence was recorded using FL1 PMT.

#### ROS detection

Oxidative stress was measured using hydroethidine (HE) (Invitrogen), which is a reduced form of ethidium bromide (EB) [21]. HE is non-fluorescent in its non-oxidized form, and it is fully permeable to the cellular membrane, thus permitting its entrance into the cytoplasm, where it competes for the available oxidants. Once HE is oxidized, fluorescent EB is trapped inside the nucleus where it intercalates into DNA. PBMCs were stimulated overnight with soluble anti-CD3 mAbs (1 μg/ml) in the presence of thimerosal at 3 μg/ml. In some experiments the anti-oxidant N-acetylcysteine (NAC) (2.5 μM) was added to the culture. Stimulated cells were co-stained with anti-CD4 mAbs, DiOC6(3) (0.1 μM) and HE (1 μM) [22] [23] and analyzed on a FACSCalibur (BD) flow cytometer.

### Functional Assays

#### T-cell proliferation

T-cell proliferation was assessed by ^3^H-thymidine incorporation or CFSE dilution assays. For ^3^H-thymidine incorporation assay, PBMCs were resuspended in culture medium (RPMI-1640 containing 2 mM L-glutamine, 20 mM Hepes, and 10 μg/ml penicillin/streptomycin) at an initial concentration of 5×10^5^ cells/200 μl per well in 96-well flat bottom plates and incubated for 5 days in the presence of the indicated antigens or vaccines. In some experiments, the plates were coated overnight at 4°C with the indicated concentrations of Panenza and washed before adding PBMCs. Cultures cells were pulsed with 1 μCi per well of [3H] thymidine (Amersham) over the final 16 h of culture. Cells were harvested on a semi-automated 96-well plate harvester (Skatron), and the amount of [3H]-thymidine incorporated was determined by liquid scintillation spectroscopy (Top count NXT™, Perkin Elmer) and expressed as cpm. In all experiments, measurements were effected on at least triplicate samples. For CFSE dilution assay, at coculture completion, CD3/CD28-stimulated CFSE-labeled T cells were harvested, costained with anti-CD4 mAb and 7-AAD, and the percentage of proliferating cells (defined as CFSElow fraction) in gated CD4+ 7-AADneg cells was determined by flow cytometry.

#### MTT cell growth assay

PBMC stimulated with CD3/CD28 mAbs, in the presence or absence of indicated concentrations of thimerosal (0.18-3 μg/ml), either overnight or in kinetics experiments, were tested for cell growth with the MTT assay kit (Millipore SAS, Molsheim, France). Absorbance was read at 570 nm.

#### Cytokine and chemokine quantification

Cytokines and chemokines were quantified in culture supernatants by multianalyte profiling (MAP) with Bio-Plex array system (Human cytokine, chemokine and growth factor assay; Biorad Life Science, Marnes-la-coquette, France), following the manufacturer's instructions. In brief, 50 μL of supernatant were incubated with antibody-linked magnetic beads for 2 h, washed twice, and incubated for 1 h with biotinylated secondary antibodies. A final incubation step of 30 min with streptavidin-phycoerythrin (PE) preceded acquisition on the Luminex 100IS.

### Immunoblot analysis

Immunoblots were performed as previously described [24]. Total cellular proteins were extracted in 250 mM NaCl-containing lysis buffer [250 mM NaCl; 0,1% NP40; 5 mM EDTA; 10 mM Na_3_VO_4_; 10 mM NaF, 5 mM DTT; 3 mM Na_4_P_2_O_7_ and the protease inhibitor cocktail (EDTA-free protease inhibitor tablets, Roche)]. 20 to 50 μg of protein extracts were run on 12% SDS-PAGE and transferred at 4°C onto a nitrocellulose membrane. After blocking, membranes were incubated with the primary antibody (cleaved caspase-3 rabbit antibody, Asp175, Cell signaling Technology, Inc) at room temperature overnight. Horseradish peroxidase-conjugated secondary anti-rabbit antibodies (Southern Biotech) were then incubated for 1 h and revealed with the enhanced ECL detection system.

### Confocal Microscopy analysis

PBMCs were incubated overnight either in medium, or with thimerosal (0.9 μg/ml or 3 μg/ml) or staurosporine (1 μg/ml). Cells were washed in PBS, fixed on poly-L-lysine coated slides (Biovalley SA, Marnes La Coquette, France), permeabilized and stained intracellularly with purified mouse anti-human cytochrome c antibody (6H2.B4, BD Pharmingen) diluted in PBA containing 0.01% saponin (Sigma Aldrich). The second-step reagent was Alexa Fluor 488 anti-mouse IgG (Invitrogen).

The nuclei were stained with DAPI nucleic acid stain (Invitrogen). The images were taken on a Leica SP5 from the Institut Pasteur Imagopole facility.

### Statistical Analyses

Parametric paired-samples Student's t-test and the ANOVA test were used to compare variables. P value <0.05 were considered statistically significant for all results.

## Results

### Dose-dependent Toxicity of Panenza on Memory T cells specific for influenza vaccine

Patients with IBD enrolled in the clinical trial MICIVAX were all vaccinated with the seasonal vaccine Mutagrip, and some of them received 21 days later the adjuvanted pandemic 2009 H1N1 vaccine (Pandemrix) or the non-adjuvanted pandemic 2009 H1N1 vaccine Panenza. PBMC from these patients were stimulated *in vitro* with various concentrations of Mutagrip or Panenza, and T-cell proliferation was analyzed at day 5. In parallel, the T-cell responses induced by persistent viruses, such as EBV and CMV, or vaccine antigens, such as TT and PPD, were analyzed on the same samples.

**Figure 1:**
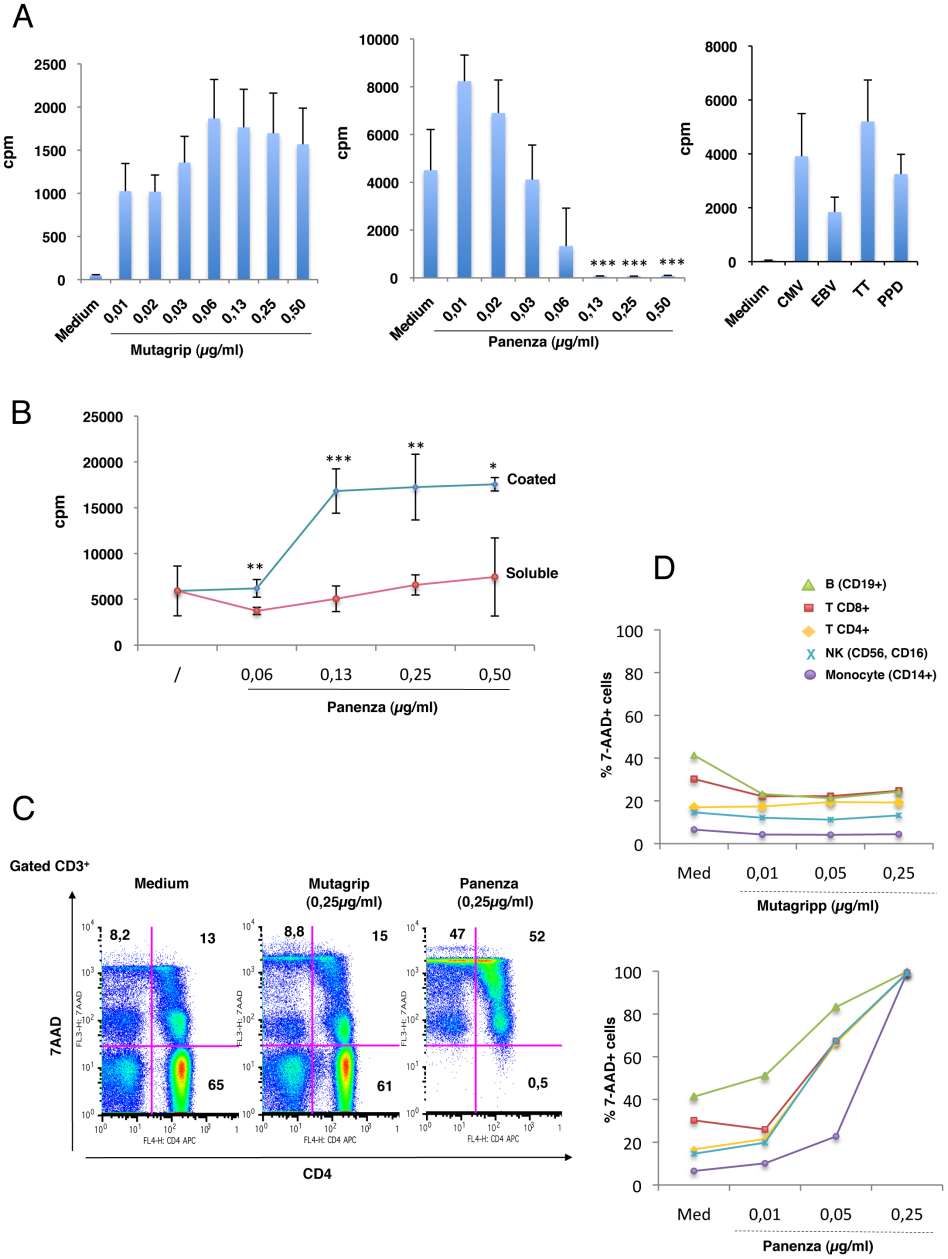
Dose-dependent Toxicity of Panenza on Memory T cells specific for influenza vaccine. A- PBMC from patients vaccinated with the seasonal flu vaccine (Mutagrip) and the pandemic 2009 H1N1 vaccine (Pandemrix) were collected 21 days after vaccine administration. Cells (5×10^5^ per well) were stimulated for 5 days with Mutagrip or the nonadjuvanted pandemic 2009 H1N1 Panenza vaccine at the indicated concentrations (vaccine concentration is expressed as the final concentration of HA). Cells were also stimulated with CMV or EBV peptides at 0.25 μg/ml, tetanus toxoid (TT) or tuberculin PPD at 5 μg/ml. Cultures were pulsed with 1 μCi per well of [3H] thymidine over the final 16 h of culture and cell proliferation expressed in cpm. All tests were done in triplicate and results show the mean values for three patients. The bars indicate the mean and standard deviation. Asterisks indicate significant *P-*values (p = 0.03) for comparison of cultures stimulated with Panenza at 0.02 to 0.5 μg/ml to cultures stimulated with 0.01 μg/ml of Panenza (ANOVA test). B- PBMC from Pandemrix vaccinated patients were incubated with soluble Panenza at indicated concentrations, or added to plates coated overnight at 4°C with indicated concentrations of Panenza. H3TdR (1μCi/well) was added at day 4 and T-cell proliferation was assessed at day 5. The bars indicate the mean and standard deviation. Asterisks indicate significant *P-*values (* <0.05, ** <0.01, ***<0.001) for comparison of cultures stimulated with coated Panenza to cultures stimulated with soluble Panenza. C- PBMC from a patient vaccinated with Mutagrip and then with Pandemrix were incubated overnight with culture medium, Mutagrip or Panenza at 0.25 μg/ml and the percentage of apoptotic cells was determined by flow cytometry combining 7-AAD staining with CD3 and CD4 membrane detection. Dot plots represent the co-expression of CD4 and 7-AAD on gated CD3^+^ T cells. D- PBMC from a patient vaccinated with Mutagrip and Panenza were incubated overnight with either vaccine at various concentrations and the percentage of apoptotic (7-AAD^+^) cells within indicated subsets was determined combining 7-AAD staining with CD19, CD3, CD8, CD4, CD56, CD16 or CD14. B cells were CD3^−^CD19^+^, T cells were CD3^+^CD8^+^ or CD3^+^ CD4^+^, NK cells were CD3^−^CD56^+^CD16^+^, monocytes were CD3^−^CD19^−^CD14^+^.

Figure 1A shows data from three patients. On the left panel, the proliferative response to Mutagrip at day 21 post-vaccination was detected at a wide range of concentrations (10 to 500 ng/ml)(vaccine concentration is expressed as the final concentration of HA) with a plateau reached at the concentration of 60 ng/ml. On the middle panel, PBMC from the same patients at day 21 post-vaccination with Panenza showed a dramatically different dose-response to this vaccine that decreased as Panenza concentration was increasing. On the right panel, the proliferative response of these patients to persistent viruses i.e. CMV and EBV, and vaccine antigens i.e. TT and PPD indicates that T cells from these patients were able to exhibit a memory response to recall antigens. This inhibitory effect of Panenza was also observed on PBMC from vaccinated healthy donors. It was therefore independent of the health condition of the donor. The inverse dose response curve to Panenza was suggestive of an in vitro toxic effect of the vaccine. To address this question, we compared the response obtained with soluble vs coated Panenza, the coating allowing attachment of vaccine proteins to the wells while eliminating non-protein molecules. Figure 1B shows data from another representative patient 21 days post-vaccination with the pandemic vaccine: a very significant memory response was detected when plates were coated with various concentrations of Panenza, reaching a plateau at 130 ng/ml, while soluble Panenza failed to induce this response. We then addressed the question of Panenza toxicity on primary lymphocytes using multiparametric flow cytometry including 7-AAD as a marker of cell death, as we previously described [19]. Overnight incubation of PBMC from a healthy donor with Panenza at 250 ng/ml induced the death of 99% of T cells, whereas the death rate induced by Mutagrip was the same as that of control cultures (21 to 23%) (Figure 1C). The toxicity of Panenza was not restricted to CD4^+^ and CD8^+^ T cells, as it affected all lymphocyte subsets, including B cells, NK cells, and monocytes, and it was detected at very low concentrations of Panenza (from 10 ng/ml) (Figure 1D). Under the same experimental conditions, Mutagrip had no toxic effect on any of these subsets (Figure 1C, 1D).

### Thimerosal is responsible for Panenza toxicity on primary T cells, and it induces mitochondrial permeability transition and caspase activation

**Figure 2:**
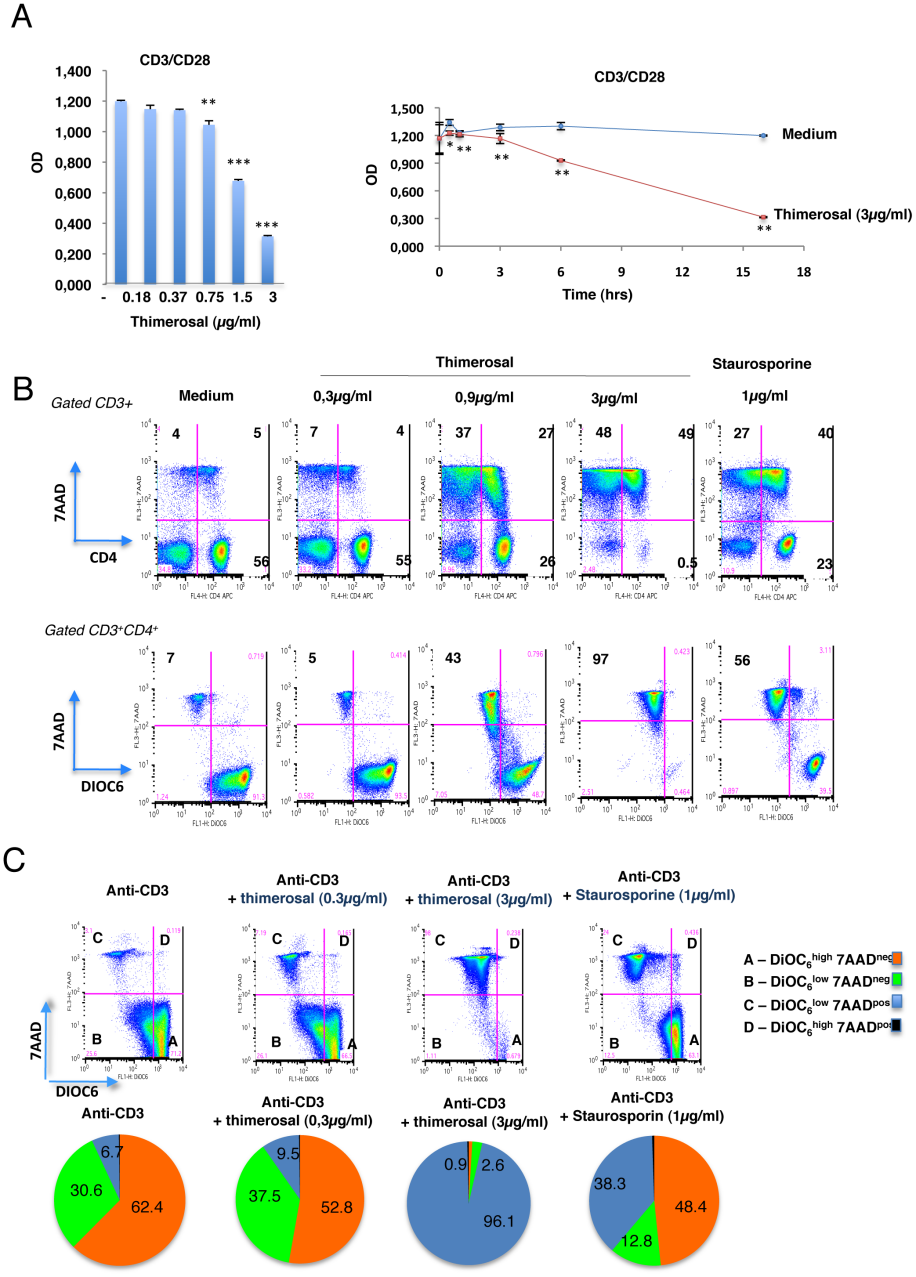
Toxicity of thimerosal on TCR-activated T cells through alterations in mitochondrial membrane potential. A- Left panel: PBMC from a HD were stimulated overnight with anti-CD3/CD28 mAbs in the presence or absence of thimerosal at indicated concentrations. Cell viability was assessed using the MTT assay. Right panel: Kinetics of thimerosal effect on the viability of PBMC from a HD activated overnight with anti-CD3/CD28 mAbs, assessed using the MTT assay. Cultures were done in triplicate and these data are representative of at least three experiments performed with three different donors. The bars indicate the mean and standard deviation. Asterisks indicate significant *p-*values (** <0.05, ***0.01, ANOVA test) for comparison of cultures stimulated in the presence of thimerosal to cultures stimulated without thimerosal. B- Induction by thimerosal of apoptotic cell death and drop in the mitochondrial transmembrane potential ΔY_m_ in unstimulated PBMC incubated overnight with the indicated concentrations of the agent. Staurosporine was used as a positive control. Apoptosis was measured with 7-AAD and variations in ΔY_m_ were assessed using the DiOC_6_(3) probe. Dot plots in the upper panel show the dose-dependent apoptotic cell death induced by thimerosal in CD3^+^CD4^+^ and CD3^+^CD4^−^ T cells, and dot plots in the lower panel show the evolution of ΔY_m_ in association with cell death on gated CD3^+^CD4^+^ T cells. C- Evolution of ΔY_m_ in association with cell death on gated CD4^+^ T cells following overnight stimulation of PBMC with anti-CD3/CD28 mAbs in the presence of thimerosal or staurosporine. The four transition states (A to D) from living cells with intact ΔY_m_ (DIOC_6_^high^ 7-AAD^neg^) to apoptotic cells (DIOC_6_^low^ 7-AAD^high^) are calculated from the dot plots and shown in pie charts. Data of one representative experiment among six are shown.

The two vaccines Mutagrip and Panenza contain 15μg of HA and NA proteins per dose, and the only difference between both is the presence of the thimerosal preservative in Panenza (45μg/dose of 0.5 ml i.e. final concentration 90 μg/ml). In order to determine the origin of the toxicity of Panenza, PBMC from a HD were stimulated 18 hrs with anti-CD3/CD28 mAbs in the presence of 0.18 to 3 μg/ml of thimerosal (equivalent dose of thimerosal present in Panenza tested at 0.06 to 1 μg/ml). Figure 2A, left panel, shows a dose-dependent toxicity of thimerosal, measured by the MTT assay, which we observed in all the HD tested (more than 20). The killing of TCR-stimulated T cells was very rapid, occurring during the first 30 minutes (Figure 2A, right panel). Thimerosal was also toxic on quiescent T cells, and the proportion of apoptotic 7-AAD^+^CD3^+^ T cells following 18 hrs of treatment increased in a dose-dependent manner, reaching 64% of CD3^+^ T cells at 0.9 μg/ml of thimerosal (Figure 2B upper panel). Staurosporine, a strong inducer of apoptosis in many cell types was used as a positive control, and the levels of apoptosis induced by thimerosal was comparable to that induced by staurosporine (Figure 2B, upper panel).

To better characterize the type of cell death induced by thimerosal on primary T cells, we used the probe DiOC_6_(3), which allows to study variations in the mitochondrial transmembrane potential ΔY_m_ during intrinsic cell death. This cyanine dye accumulates in the mitochondria matrix under the influence of ΔY_m_ [25]. As shown in Figure 2B lower panel, T-cell apoptosis induced by 18-hr incubation with thimerosal was associated with a drop in ΔY_m_ in CD3^+^CD4^+^ T cells, which was observed exclusively in 7-AAD^+^ T cells. As a positive control, staurosporine also induced ΔY_m_ disruption in CD3^+^CD4^+^T cells, in agreement with previous studies in human PBL [26] or in Hela cells [27]. The involvement of the mitochondrial pathway in thimerosal induced T-cell death was also observed when T cells were stimulated through the TCR. The impact of thimerosal on the transition states from living cells with intact ΔY_m_ (DIOC_6_^high^ 7-AAD^neg^) to still living cells with a drop in ΔY_m_ (7-AAD^neg^ DIOC_6_^low^ cells) to dead cells (DIOC_6_^low^ 7-AAD^high^) is shown in a representative experiment (Figure 2C).

**Figure 3:**
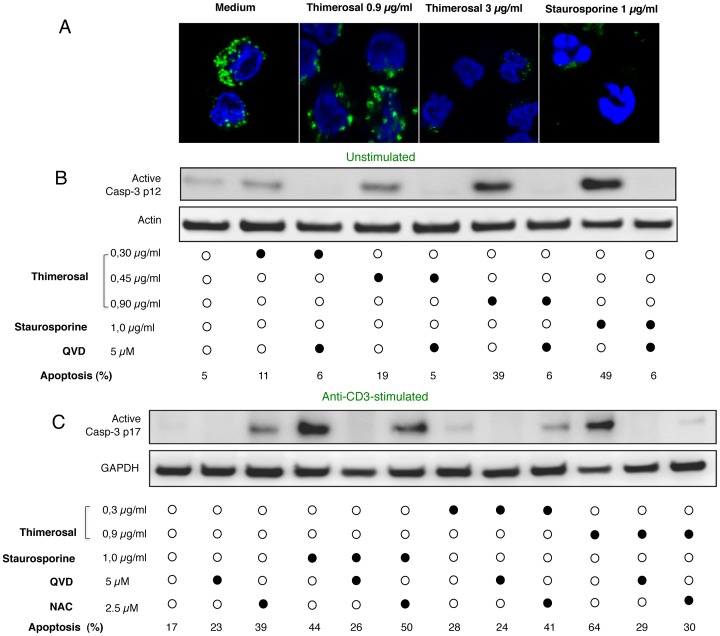
Thimerosal induces cytochrome c release and caspase-3 activation. A- Confocal analysis of PBMC incubated 16 hours either in the presence of thimerosal (0.9 or 3 μg/ml), or with staurosporine (1 μg/ml) as a positive control. Cells were processed for cytochrome c staining (green) and co-stained with DAPI (blue) to detect nuclei modifications. B-C Western blot analysis of active caspase-3 expression in PBMC incubated for 16 h in medium (unstimulated) (B) or stimulated with anti-CD3 mAbs (C). The impact of indicated concentrations of thimerosal or staurosporine on active caspase-3 expression is shown, together with the influence of the broad caspase inhibitor QVD and the anti-oxidant NAC.

In addition to the loss of ΔY_m_, overnight treatment of primary T cells with thimerosal induced cytochrome c release from the mitochondria, as shown by confocal microscopy (Figure 3A). The labeled cytochrome c was restricted to mitochondria in control cells incubated in medium, while a more diffuse labeling dispersed in the cytoplasm was observed in the presence of 0.9 μg/ml of thimerosal. In cells treated with 3 μg/ml of thimerosal, cytochrome c was washed out from the cells after its release and no more labeled cytochrome c was detected. Same picture was seen in staurosporine-treated cells (Figure 3A). The nuclei were condensed but showed a strange jagged morphology following the treatment with 3 μg/ml of thimerosal, while they were fragmented after staurosporine treatment (Figure 3A).

The biochemical manifestations of the induction of the mitochondrial permeability transition (MPT) were associated with caspase-3 activation. This was observed whether T cells were unstimulated (Figure 3B) or stimulated with anti-CD3 mAbs (Figure 3C). Western blot analysis showed that thimerosal activated caspase -3 in both unstimulated and TCR-triggered T cells in a concentration-dependent manner. To confirm the role of caspase activation in thimerosal-induced apoptosis, the broad caspase inhibitor QVD [28] was used. T cells were treated with QVD (5μM) and thimerosal (from 300 to 900 ng/ml) overnight. Figure 3 B, C show that QVD prevented activation of caspase-3 induced by increasing doses of thimerosal, and this was associated with the prevention of T-cell apoptosis. Similar data were obtained when unstimulated or stimulated T cells were treated with staurosporine (Figure 3B, C). These results indicate that induction of MPT and cleavage activation of caspase-3 might play an important role in apoptosis induced by thimerosal in human primary T cells, either non activated or activated following TCR ligation.

### Inhibition of ROS prevents thimerosal-induced apoptosis of TCR-stimulated T cells

**Figure 4:**
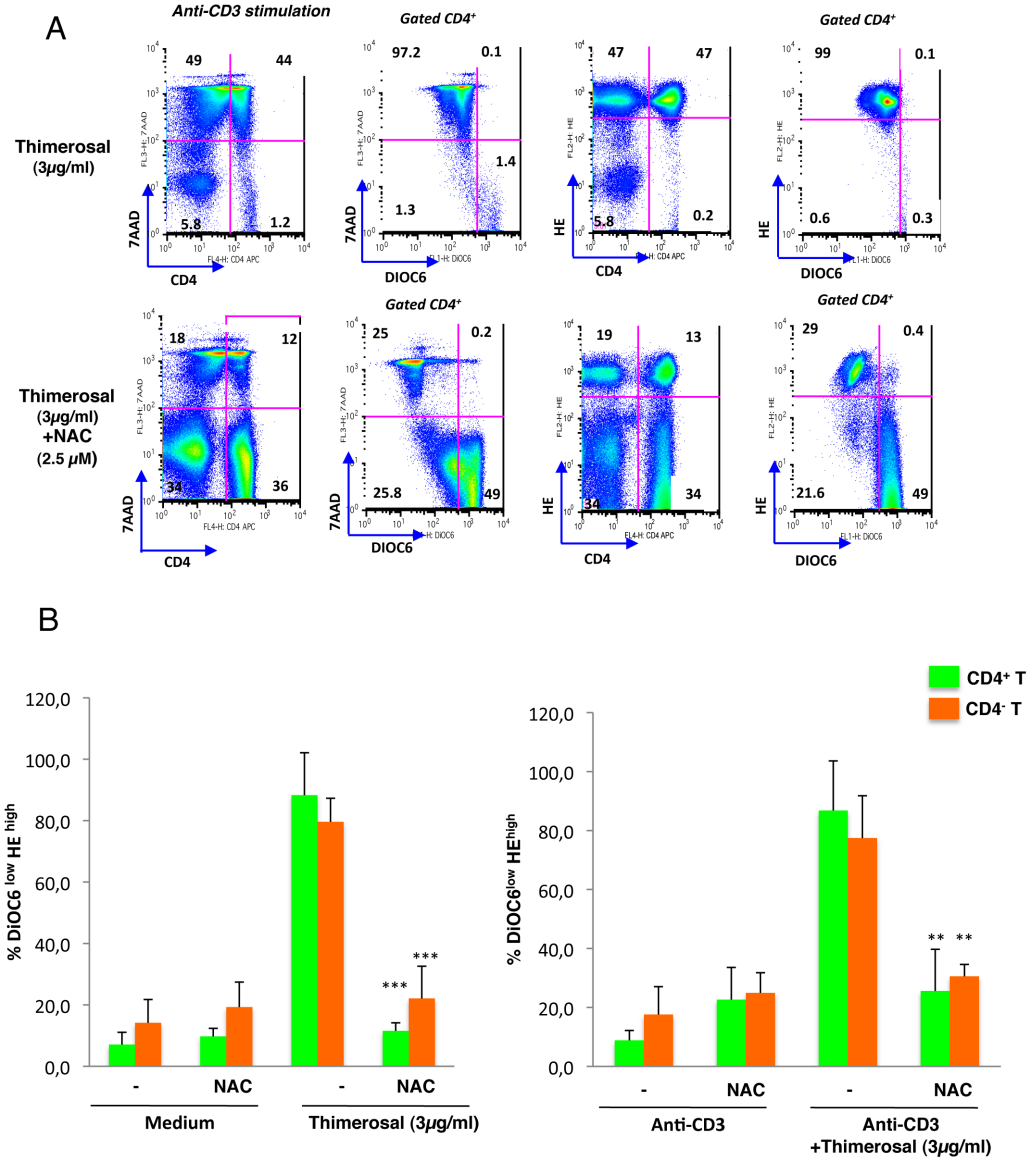
ROS induction by Thimerosal in TCR-activated T cells and anti-apoptotic effect of NAC. A- PBMC from a HD were stimulated overnight with anti-CD3 mAbs in the presence of thimerosal at 3 μg/ml, and NAC (2.5 μM) was added in half of the cultures. ROS production was detected with HE, ΔY_m_ was assessed using the DiOC_6_(3) probe, and apoptosis was measured with 7-AAD. The dot plots show combined analysis of ΔY_m_ and apoptosis or ΔY_m_ and ROS production on gated CD4^+^ T cells, in a representative experiment out of three experiments performed with three different HD. B- PBMC from a HD were either unstimulated (left panel) or stimulated overnight with anti-CD3 mAbs in the presence or absence of thimerosal at 3 μg/ml (right panel). The anti-oxidant NAC (2.5 μM) was added in some cultures. The percentages of CD4^+^ and CD4^−^ T cells with a concomitant drop in ΔY_m_ and increased oxidizing ability (DIOC_6_^high^ 7-AAD^neg^) under the indicated culture conditions are shown. The bars indicate the mean and standard deviation from 3 experiments with three different donors. Asterisks indicate significant *P-*values (** <0.001, ***0.0001) for comparison of cultures stimulated with thimerosal and NAC to cultures stimulated with thimerosal only.

We then addressed the question of the possible involvement of oxidative stress in thimerosal-induced apoptosis, since a temporal association between mitochondrial membrane permeabilization and ROS production was reported in several cell types such as primary thymocytes or tumor cells [22], [29] [30]. We used HE to detect cells with an increased oxidizing ability that we combined with DiOC6_s_ to make the link between thimerosal-induced ΔY_m_ disruption and oxidative stress. Figure 4A shows that thimerosal at 3 μg/ml induced a high production of ROS in anti-CD3-activated T cells, and all HE^+^ cells showed a drop in ΔY_m_. To confirm the role of ROS in thimerosal-induced apoptosis, the antioxidant NAC was added together with thimerosal at the initiation of the overnight stimulation of PBMC with anti-CD3 mAbs. As shown in Fig 4A,B the inhibition of ROS levels by NAC prevented ΔY_m_ disruption and remarkably inhibited the proapoptotic effect of thimerosal in both CD4^+^ and CD4^-^ T cells. This protective effect of NAC was observed whether T cells were stimulated by anti-CD3 mAbs or unstimulated. Besides, NAC decreased the level of cleavage activation of caspase-3 after exposure of CD3-stimulated T cells to different doses of thimerosal, as shown in Figure 3C. NAC had a similar effect on staurosporine-induced caspase-3 activation (Figure 3C).

### Non-toxic concentrations of thimerosal significantly inhibit the proliferation of T cells following TCR ligation, resulting in G0/G1 phase arrest

**Figure 5:**
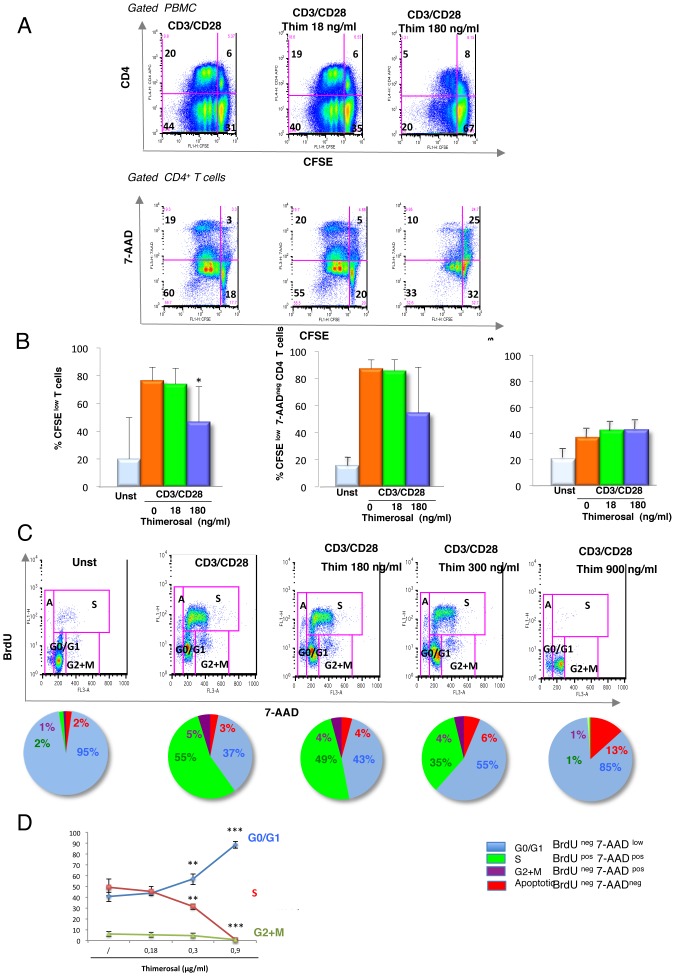
Thimerosal inhibits T-cell proliferation and induces a G0/G1 cell cycle arrest. A- PBMC from a HD were labeled with CFSE and stimulated during 4 days with anti-CD3/CD28 mAbs in the presence or not of thimerosal. At the end of the culture, cells were co-stained with anti-CD4 mAbs and 7-AAD, and the rate of proliferation in CD4 T cells was determined (upper panel) combined with their priming for apoptosis (lower panel). B- The experiment of panel A was performed with 7 different HD and the bars indicate the mean and standard deviation. The left panel shows the percentage of total proliferating cells, the middle panel shows the proliferation of living cells and the right panel shows the proliferation of dying cells. Asterisks indicate significant *P-*values (* <0.05, ANOVA test) for comparison of cultures stimulated in the presence of thimerosal at 180 ng/ml to cultures stimulated in the absence of thimerosal. C: Cell cycle analysis of PBMC stimulated with anti-CD3/CD28 mAbs in the presence of thimerosal at three different concentrations. The pie charts show the repartition of cells in the various phases of the cell cycle. D- Curves show the mean values from 7 HD, demonstrating the accumulation of cells in G0/G1 phase and the disappearance of cells in the S phase after exposure to increasing concentrations of thimerosal. Asterisks indicate significant *P-*values (** <0.01, ***0.001) for comparison of the proportions of cells in G0/G1 or in S phase with or without thimerosal.

The effect of thimerosal at non-toxic concentrations on the proliferation of T cells in response to CD3/CD28 ligation was determined. To quantitatively analyze the proliferation and apoptosis of primary T cells *ex-vivo*, we labeled PBMC with CFSE, a fluorochrome whose per cell fluorescent intensity halves with each round of cell proliferation [31]. After 4 days of stimulation with CD3/CD28 mAbs, cells were stained with CD4-specific antibodies and 7-AAD. Figure 5A shows in a representative experiment that thimerosal treatment at 180 ng/ml resulted in inhibition of cell proliferation by more than 50% in both CD4^+^ and CD4^−^ T cells, as evidenced by the drop in the percentage of CFSE^low^ cells (a reduction of 64% in control cultures to 25% in cultures with thimerosal). This inhibition of cell proliferation was not associated with activation-induced cell death, as shown by the dot plots combining 7-AAD/CFSE staining in gated CD4^+^ cells (Figure 5A). Indeed, 25% of CD4^+^CFSE^low^ cells were 7-AAD^+^ in CD3/CD28 stimulated control cultures compared to 23% in cultures containing thimerosal at 180 ng/ml (Fig 5A). Figure 5B shows a synthesis of the data obtained with 7 donors, indicating that exposure to thimerosal decreases the percentage of living proliferating cells (CFSE^low^/7-AAD^neg^ cells) (middle panel) while it does not increase the percentage of dead proliferating cells (CFSE^low^/7-AAD^+^ cells) (right panel).

This inhibition of proliferation could thus be due to cell cycle arrest. Using the BrdU assay, which combines BrdU incorporation into newly synthesized DNA strands to the DNA dye 7-AAD, we found that exposure to thimerosal resulted in the accumulation of T cells in the G0/G1 phase of the cell cycle (Figure 5C and 5D). Cell cycle analysis performed after 72-hour incubation of CD3/CD28-stimulated PBMC with increasing concentrations of thimerosal (180, 300 and 900 ng/ml) showed that T cell-accumulation in the G0/G1 phase occurred in a dose-dependent manner, and after exposure to 900 ng/ml of thimerosal, only 1% of the cells were in S phase compared to 55% in stimulated control cultures (Figure 5C and 5D). As already shown in CFSE experiments, cell cycle arrest was not associated with significant increase in apoptotic cells (Figure 5C).

### Impact of thimerosal on cytokine and chemokine release upon TCR ligation

**Figure 6:**
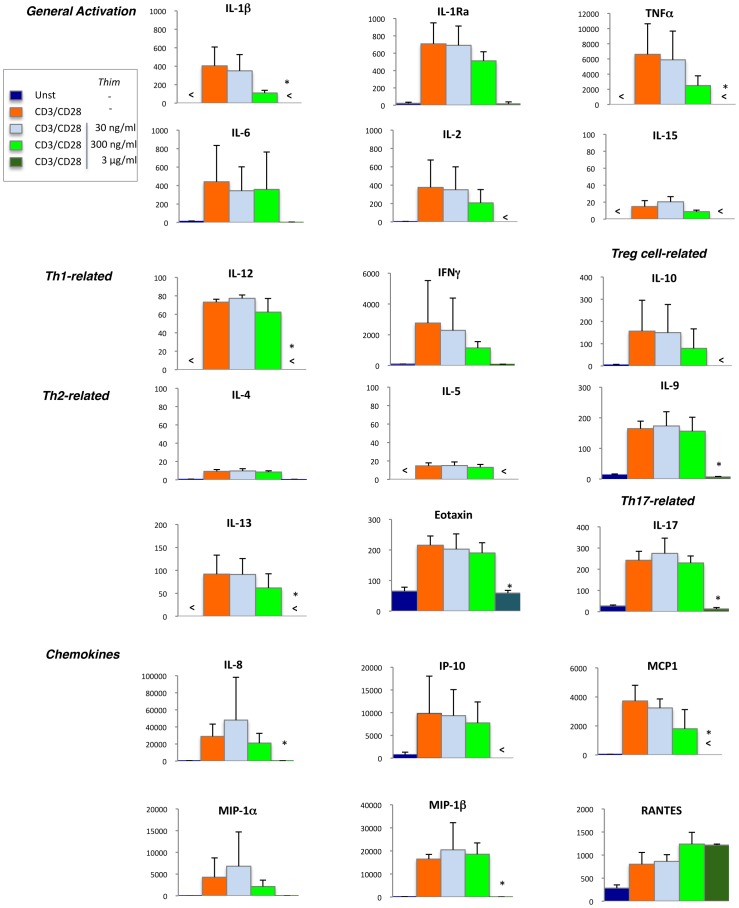
Impact of thimerosal on cytokine and chemokine release upon TCR ligation. Pattern of cytokines and chemokines simultaneously quantified by multiplex bead assay arrays in supernatants from freshly isolated PBMC following 16-hour stimulation with anti-CD3/CD28 mAbs in the absence or presence of three different concentrations of thimerosal. The bars indicate the mean and standard deviation from experiments with three different donors. Asterisks indicate significant *P-*values (* <0.05, ANOVA test) for comparison with cultures without thimerosal.

Fig 6 shows the pattern of cytokines and chemokines simultaneously quantified by multiplex bead assay arrays in supernatants from 16-hour stimulation with anti-CD3/CD28 mAbs of freshly isolated PBMC from HD. High levels of cytokines associated with general activation, such as IL-1β, TNF alpha, IL-6 and IL-2 were detected following TCR ligation. As expected, the Th1 cytokine IFN gamma was produced in high amount whereas low levels of Th2 cytokines (IL-4, IL-5 or IL-13) were detected. Cytokines involved in the promotion of Th17 such as IL-9 and IL-17 were significantly produced, as well as the anti-inflammatory cytokine IL-10. Finally, extremely high levels of the chemokines MIP1-alpha, MIP1-beta, IP-10, MCP1, IL-8 and RANTES were induced by CD3/CD28 ligation.

Following exposure to various concentrations of thimerosal (30, 300 and 3000 ng/ml), the release of the proinflammatory cytokines IL-1β, TNF alpha, IL-2 and the Th1 cytokine IFN gamma was reduced in a dose-dependent manner. The observation of the concomitant decrease in IL-1β and IFN gamma production is consistent with the recent finding that IFN gamma potentiates IL-1β release from human cells [32]. The levels of most of the cytokines were under the threshold of detection after exposure to Thimerosal 3μg/ml, which may be partly due to the toxicity of the agent at this concentration. Exposure to thimerosal did not induce the release of the Th2 cytokines IL-4 and IL-5, and it slightly inhibited the production of IL-13, indicating that thimerosal did not induce a Th1 to Th2 shift. Th17-related cytokines were not affected, whereas an inhibition of IL-10 production was found. Among the chemokines released following TCR ligation, MCP1 was the most susceptible to the suppressive effect of thimerosal in a dose-dependent manner. It is noteworthy that RANTES production was increased at the dose of 300 ng/ml of thimerosal and it persisted at high level after exposure to 3 μg/ml of the agent. Altogether, these data indicate that exposure of T cells to non-toxic concentrations of thimerosal during their activation through the TCR decreases the pro-inflammatory response, including the production of IFN gamma essential in mediating protective immunity to viral infections.

## Discussion

This study was initiated when we discovered, in a proliferative assay, the *ex-vivo* dose-dependent toxicity of the multidose non-adjuvanted pandemic 2009 H1N1 vaccine Panenza on PBMC from vaccinated people, while the seasonal influenza vaccine Mutagrip was not toxic under the same conditions. Coating of Panenza proteins on the plates before lymphocyte stimulation enabled the detection of Flu-specific memory T cells, indicating that the toxic molecule was not a protein. Comparison of Mutagrip and Panenza composition revealed that thimerosal was the only compound that differed between the two vaccines. As a vaccine preservative, thimerosal is used in concentrations of 0.003% to 0.01%. [2]. A vaccine containing 0.01% thimerosal contains 50 μg of thimerosal per 0.5 ml dose or approximately 25 micrograms of mercury per 0.5 ml dose. Panenza that we used in our study contained 45 μg of thimerosal per 0.5 ml dose (90 μg/ml). The toxic and immunomodulatory effects of thimerosal that we report herein have been detected at the final concentration of 30 ng/ml to 3 μg/ml, i.e. 3000 to 30 times less concentrated than in the vaccine. Therefore our results provide meaningful information on the possible effects of the use of thimerosal on vaccine-induced immunity. Our study showed that short-term exposure to thimerosal at low concentrations could induce mitochondrial apoptosis in TCR-activated primary human T cells and could suppress Th1 polarization at non-toxic concentrations. These observations suggest that thimerosal may alter *in vivo* vaccine-induced immunity. Moreover, it was found to induce *ex-vivo* false-positive results in the monitoring with Elispot technique of the clinical efficacy of influenza vaccines, as we reported recently[33].

Although thimerosal has been shown to promote apoptosis in some cell types, they were all tumor cells [34] [8], [9], [10], [11], [12], [15], [35], [36] and this study is the first one to demonstrate the proapoptotic effect of thimerosal on primary human T cells. The dose-dependent toxic effect of thimerosal was detected after a short-term (16 h) exposure to the agent in both unstimulated and TCR-stimulated CD4 and CD8 T cells, but also in other blood mononuclear subsets such as NK cells, monocytes or B cells, indicating a broad effect of the agent on the effectors of the immune system. In the first 3 hours of exposure to thimerosal of primary lymphocytes from healthy donors, morphological changes, including membrane alterations and cell shrinkage, were observed, detected by the FSC/SSC parameters by flow cytometry. Cell viability, assessed by the MTT assay, as well as the 7-AAD flow cytometry assay, showed a time- and concentration-dependent decrease in cell survival upon thimerosal exposure. Mitochondria play a crucial role in regulating apoptosis [15] [37], and it was found involved in thimerosal-induced cell death of several tumor cell types [10] [9] [12] [11] [15]. To identify the apoptotic pathway associated with thimerosal-mediated cell death of primary lymphocytes, we used the probe DiOC_6_(3), which allows to study variations in the mitochondrial transmembrane potential ΔY_m_ during intrinsic cell death [25]. We found that thimerosal induced the loss of mitochondrial membrane potential both in quiescent T cells and in TCR-activated T cells. This was strongly associated with enhanced intracellular reactive oxygen species (detected with HE), release of cytochrome c and caspase-3 activation. Interestingly, thimerosal toxicity was completely prevented by the radical scavenger NAC. Altogether, these data are consistent with previous observations made in other cell types, such as Jurkat T cells where thimerosal triggered apoptosis via the mitochondrial pathway by inducing oxidative stress and depletion of GSH [15], and HeLa S epithelial cells in which thimerosal-mediated toxic response was almost completely suppressed by pretreating the cells with NAC [11].

We also report that Thimerosal was a potent inhibitor, at non-toxic concentrations, of T-cell proliferation induced in a 3-day culture by TCR ligation with anti-CD3/CD28 mAbs, as shown by the combined staining with CFSE/7-AAD. Cell cycle analysis performed under these conditions with increasing concentrations of thimerosal (180 to 900 ng/ml) revealed a dose-dependent accumulation of T cells in G0/G1 phase, and after exposure to 900 ng/ml of thimerosal, only 1% of the cells were in S phase. Woo *et al* reported that thimerosal induced G2/M phase arrest in human leukemic cells [36] and Li *et al* reported that thimerosal induced S phase arrest in C2C12 myoblast cells [12]. Our study is the first to report that thimerosal induces a G0/G1 arrest. This suggests that thimerosal may cause cell cycle arrest by different mechanisms in different cell types, and that thimerosal may interfere with TCR-dependent T-cell activation at low and non-toxic concentrations.

Although antibodies are the predominant protective correlates in the case of vaccines, cell-mediated immune functions are critical in protection against intracellular infections, and in almost all diseases, CD4^+^ cells are necessary to help B cell development [38]. For example, McElhaney et al. [39] found that cytokine production and proliferation of T cells in the presence of influenza antigen correlated with protection of elderly adults. Thus, whereas antibody production is critical in the young to prevent primary influenza infection, CD4^+^ cells may be more important in immunologically experienced individuals undergoing heterosubtypic infection. This was recently suggested in a study conducted in healthy volunteers with no detectable antibodies to the challenge viruses H3N2 or H1N1, demonstrating that preexisting CD4 T cells responding to pandemic H1N1 internal proteins with cytotoxic activity were associated with lower virus shedding and less severe illness [40]. The authors suggested that these cells are an important correlate of homotypic and heterotypic response that may limit severity of influenza infection by new strains in the absence of specific antibody response [40]. In another study, higher frequency of preexisting IFN-γ^+^IL-2^-^ CD8^+^ T cells specific to conserved core protein epitopes inversely correlated with symptom score, and correlated with crossprotection against influenza in the absence of crossreactive neutralizing antibodies [41]. In this context, our observations of the ability of thimerosal to inhibit T-cell proliferation in response to influenza vaccine antigens are meaningful. In particular, we report that non-toxic concentrations of this agent had an impact on the pattern of mediators produced in response to CD3/CD28 mAbs, as thimerosal at 300 ng/ml inhibited the production by activated T cells of the proinflammatory molecules IFN-γ, IL-1β and TNF-α. The Th1 cytokine IFN-γ is critical for innate and adaptive immunity against viral and intracellular bacterial infections. IFN-γ contributes to macrophage activation by increasing phagocytosis and priming the production of proinflammatory cytokines and potent antimicrobials, it also controls the differentiation of naive CD4 T cells into Th1 effectors, which mediate cellular immunity against viral and intracellular pathogens [42]. Inhibition of IFN-γ production following exposure to thimerosal may be detrimental for vaccine-specific immunity if this effect occurred *in vivo*, as suggested by the essential role of T cells producing this cytokine in protection against illness in influenza antibody naïve individuals [41]. Of note, thimerosal did not induce a shift to Th2 cytokine production, in contrast to the finding of an earlier study[14]. However, the experimental conditions were different since a prior “priming” of mature DC with thimerosal was required for Th2 differentiation in DC-T cocultures[14].

In conclusion, our work indicates that *ex-vivo* exposure of quiescent or TCR-activated primary human T cells to thimerosal induces apoptosis associated with depolarization of mitochondrial membrane, generation of reactive oxygen species, release of cytochrome c from the mitochondria and caspase-3 activation. Moreover, exposure to non-toxic concentrations of thimerosal induces cell cycle arrest in G0/G1 phase and inhibition of the release of proinflammatory cytokines, including IFN-γ. Multi-dose vials of vaccines containing thimerosal remain important for vaccine delivery, in particular in the event of pandemics or health emergencies. However, our observations regarding its *ex-vivo* toxicity and immunomodulatory effects on human primary lymphocytes highlight the need to use this preservative with caution and avoid it if possible.
